# Bioinformatics analyses combined microarray identify the deregulated microRNAs in oral cancer

**DOI:** 10.3892/ol.2014.2070

**Published:** 2014-04-15

**Authors:** JING CUI, DALU LI, WENMEI ZHANG, LIANG SHEN, XIN XU

**Affiliations:** 1 Department of Oral and Maxillofacial Surgery, Jinan Stomatologic Hospital, Jinan, Shandong 250010, P.R. China; 2 School of Stomatology, Shandong University, Jinan, Shandong 250010, P.R. China; 3 Department of Oral Surgery, Jinan Stomatological Hospital, Jinan, Shandong 250010, P.R. China; 4 Department of Obstetrics and Gynecology, Shandong Provincial Hospital Affiliated to Shandong University, Jinan, Shandong 250010, P.R. China

**Keywords:** microRNA, oral cancer, hsa-miR-15a

## Abstract

MicroRNAs (miRNAs) are important in the regulation of cell growth, differentiation, apoptosis and carcinogenesis. The overexpression of oncogenic miRNAs or the underexpression of tumor suppressor miRNAs exhibits a critical function in the tumorigenesis of oral cancer. The aim of the present study was to identify differentially expressed miRNAs (DE-miRNAs), which may differentiate oral cancer from normal tissues, as well as the molecular signatures that differ in tumor histology. The miRNA expression profiles of GSE28100 [the Gene Expression Omnibus (GEO) accession number] were downloaded from the GEO database and an independent sample t-test was used to identify statistical differences between the DE-miRNAs of the oral cancer patients and the healthy control subjects. The target genes of DE-miRNA were retrieved from the miRecords database. Furthermore, a protein-protein interaction network was constructed using the Search Tools for the Retrieval of Interacting Genes database and Cytoscape software. A total of 15 DE-miRNAs were identified and among them, hsa-miR-15a drew specific attention. Gene Ontology analysis revealed that the target genes of fibroblast growth factor (FGF)2 are involved in the progression of oral cancer. Furthermore, functional analysis indicated that the FGF-receptor signaling pathway was significantly upregulated in oral cancer. hsa-miR-15a is important in the regulation of oral cancer and thus, may present a potential biomarker for the prediction of oral cancer progression.

## Introduction

Oral cancer is the sixth most common cancer worldwide, with a high prevalence in regions where individuals habitually smoke cigarettes and consume alcohol (1). In addition, the five-year relative survival rate of distant metastasis for oral cancer is ~30–40% (2). In the USA, ~41,380 individuals are diagnosed with oral cancer annually and ~7,890 individuals succumbed to the disease in 2012 (3).

MicroRNAs (miRNAs) are a family of endogenous, non-coding, 22–25 nt RNAs that regulate target mRNA. Accumulating evidence indicates that miRNAs are involved in important biological processes associated with apoptosis, proliferation, differentiation, angiogenesis and metastasis. Therefore, the deregulation of such processes may exhibit an effect on cancer initiation, progression and treatment outcome (4,5).

It is hypothesized that miRNAs may serve as valuable tools in cancer diagnosis. Previous studies using miRNA microarray analysis have identified statistically unique profiles, which may discriminate cancer samples from healthy control samples (6). Siow *et al* (7) used an miRNA microarray to identify the differentially expressed miRNAs (DE-miRNAs) between oral squamous cell carcinoma and non-cancer cells, and miR-31 and miR-375 were found to significantly correlate with clinicopathological parameters.

The aim of the present study was to identify the miRNAs, which may be important in the progression of oral cancer and to analyze their involvement in this process. An independent sample t-test was used to analyze the raw data in order to obtain credible data of the DE-miRNAs. In addition, an interaction network was constructed using the Search Tools for the Retrieval of Interacting Genes (STRING) database and Cytoscape software. The results of the current study support the hypothesis that miRNA expression is deregulated in oral cancer patients compared with healthy individuals.

## Materials and methods

### Microarray analysis

The miRNA expression profile of GSE28100 [the Gene Expression Omnibus (GEO) accession number] was downloaded from the GEO database, which was collected by Jung *et al* (8). The expression data of miRNAs was obtained using the GEO accession number, GSE28100, with the purpose of identifying aberrantly expressed miRNAs in oral squamous cell carcinomas. The expression profiles of miRNAs in 17 patients with oral cancer and three healthy control subjects were available.

### Identification of DE-miRNAs

The raw data were transformed into identifiable expression data and the missing data was completed. Background corrections and quartile data normalization were performed with the robust multi-array average using the default parameters in the affy package. In addition, the data were analyzed using BRB-ArrayTools version 4.2 (National Cancer Institute; http://linus.nci.nih.gov/BRB-ArrayTools.html).

### Predicting the target genes of DE-miRNAs

The miRecords database (http://miRecords.umn.edu/miRecords), which is a resource for animal miRNA-target interactions, was used to analyze the target genes of the DE-miRNAs. miRecords integrates the predicted targets of the following miRNA target prediction tools: DIANA-microT (http://diana.csla-b.ece.ntua.gr/microT), MicroInspector (http://bioinfo.uni-plovdiv.bg/microinspector), miRanda (http://www.microrna.org/microrna/home.do), MirTarget2 (http://mirdb.org/miRDB), miTarget™ (http://cbit.snu.ac.kr/~miTarget), NBmiRTar (http://wotan.wistar.upenn.edu/NBmiRTar/login.php), PicTar (http://pictar.bio.nyu.edu), PITA (http://genie.weizmann.ac.il/index.html), RNA22 (http:/cbcsrv.watson.ibm.com/rna22.html), RNAhybrid (http://bibiserv.techfak.uni-bielefeld.de/rnahybrid) and TargetScan (http://www.targetscan.org). The genes that were predicted by at least five of the 10 databases were selected as DE-miRNA targets for subsequent analysis to reduce the quantity of false-positive results.

### Network analysis and functional annotation

The STRING database includes experimental and predicted interaction information and STRING version 9.1 comprises of >1,100 completely sequenced organisms (9). To identify the interactive associations between the target genes and other genes, the target genes of DE-miRNAs were input into STRING. The Cytoscape software was used to visualize these associations and the mined modules.

The Database for Annotation, Visualization and Integrated Discovery (DAVID) includes a broad selection of functional annotation tools for understanding the biological significance of numerous genes. In the current study, DAVID was used to label the function of genes within the modules and the Gene Ontology (GO) terms with an adjusted P-value of <0.05 and a count >2 were selected.

### Statistical analysis

An independent sample t-test was used to identify the DE-miRNAs between the oral cancer patients and healthy control subjects, and P<0.001 was considered to indicate a statistically significant difference.

## Results

### Identification of DE-miRNAs

miRNA expression data was obtained using the accession number, GSE28100, and included 17 patients with oral cancers and three healthy control subjects. The miRNA expression data was analyzed by BRB-ArrayTools and 15 miRNAs exhibited significant differential expression (P<0.001; Table I).

### Target gene prediction

Since miRNAs regulate the post-transcriptional regression of target genes, the putative target genes of DE-miRNAs were retrieved from miRecords, which selects the target genes that have been retrieved by at least five databases. Furthermore, these target genes were searched for using PubMed and a large list of target genes were confirmed to be associated with oral cancer (Table II). Among these genes, hsa-miR-15a had the greatest number of target genes associated with oral cancer.

### Interaction network construction and module analysis

**Figure 1:**
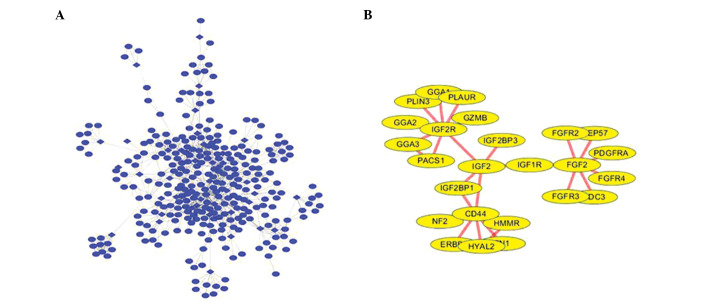
(A) PPI network construction. (B) The module identified from the PPI network. PPI, protein-protein interaction; PLAUR, plasminogen activator urokinase receptor; GGA, golgi-associated, γ adaptin ear containing, ARF binding protein; PLIN3, perilipin 3; IGF2R, insulin-like growth factor 2 receptor; GZMB, granzyme B; PACS1, phosphofurin acidic cluster sorting protein 1; IGF2BP, insulin-like growth factor 2 mRNA binding protein; IGF2, insulin-like growth factor 2; NF2, neurofibromin 2; HYAL2, hyaluronoglucosaminidase 2; HMMR, hyaluronan-mediated motility receptor; IGF1R, insulin-like growth factor 1 receptor; FGF2, fibroblast growth factor 2; FGFR, fibroblast growth factor receptor; PDGFRA, platelet-derived growth factor receptor, α polypeptide.

The target genes of 12 DE-miRNAs were input into the STRING database, which identified the significant interactions with a confidence score of >0.9. In addition, a protein-protein interaction (PPI) network was constructed using Cytoscape software (Fig. 1).

The PPI network reveals the molecular mechanisms of oral cancer, however, it contains numerous nodes and interactions, which makes it difficult to select the useful information. Therefore, the modules were mined in the PPI network to include insulin-like growth factor (IGF)2-receptor (R), cluster of differentiation 44, IGF2, IGF1-R and fibroblast growth factor (FGF)2. Functional analysis demonstrated that the genes in this module could be divided into 21 functional GO terms (the 10 most significant terms are shown in Table III). Among these functional nodes, the most significant GO category was identified to be the FGF-R signaling pathway.

The target genes of FGF2 were identified to be associated with the GO categories of apoptosis, programmed cell death, cell migration, cell death and cell motility.

## Discussion

In the present study, 15 DE-miRNAs were identified to exhibit a regulatory function in the progression of oral cancer. As a result of retrieving the target genes of the DE-miRNAs from miRecords, the target genes of 12 DE-miRNAs were found to be associated with oral cancer. Through PPI network construction and module analysis, an FGF2 module was formed and was identified to be significant in the progression of oral cancer. Furthermore, functional analysis showed that the module was significantly associated with the FGF-R signaling pathway.

The expression of 12 DE-miRNAs, including hsa-miR-15a, was identified as a possible biomarker to monitor oral cancer progression and early diagnosis. In addition, hsa-miR-15a was found to be downregulated in certain hematological tumors and is considered to regulate cancer-associated genes that influence apoptosis, the cell cycle, proliferation and survival (10,11). In addition, hsa-miR-15a is frequently downregulated in chronic lymphocytic leukemia, prostate cancer and non-small cell lung cancer (12–14), and the inhibition of hsa-miR-15a significantly increases the secreted matrix metalloproteinase-9 expression in neuroblastoma (15). However, Ricieri *et al* (16) identified that hsa-miR-15a expression levels were upregulated in the majority of oral cancers samples. The results of the current study using the accession number, GSE28100 revealed a 3.58-fold change in hsa-miR-15a expression, compared with that of the healthy control subjects, which indicated that hsa-miR-15a is involved in the progression of oral cancer.

In addition, FGF2 formed a module in the PPI network that was constructed based on oral cancer samples, which indicated that FGF2 has an important function in the progression of oral cancer. FGF2 is an 18-kDa non-glycosylated polypeptide consisting of 146 amino acids (17), which mediates various cellular events, including migration, angiogenesis, motility, proliferation and differentiation (18,19).

In addition, FGF2 promotes tumor progression and previous studies indicate that the upregulation of FGF2 is important in prostate carcinogenesis and malignant progression (20). FGF2 is one of the most well-studied factors involved in angiogenesis (21). Lau *et al* (22) identified that the expression of FGF2 decreases E-cadherin levels by upregulating its transcriptional repressors, Slug and ZEB1, in human ovarian cancer cells.

The FGF-R, a sub-family of the superfamily of receptor tyrosine kinases, may regulate human development and metabolism. Previous studies have shown that FGF-R may be important in carcinogenesis (23,24). Furthermore, studies have indicated that FGF-R1 is amplified in 20% of squamous non-small cell lung cancers (25) and mutations of FGF-R2 have been described in 12% of endometrial carcinomas (26). Furthermore, ~10% of gastric cancer cases exhibit FGF-R2 amplification and mutations (27).

In conclusion, the current study identified 15 DE-miRNAs, which may be important in the progression of oral cancer and hsa-miR-15a demonstrated the greatest quantity of target genes. In addition, FGF2 expression was identified to be significantly associated with the presentation of oral cancer. However, further investigation regarding the function of FGF2 is required.

## Figures and Tables

**Table I:** Differentially expressed miRNAs obtained using Gene Expression Omnibus accession number, GSE28100.

n	miRNA	Fold change	P-value
1	hsa-miR-424	10.31	0.0000650
2	hsa-miR-21	6.34	0.0002210
3	hsa-miR-15b	7.81	0.0006760
4	hsa-miR-923	0.21	0.0001064
5	hsa-miR-146b-5p	5.54	0.0001082
6	hsa-miR-331-3p	2.92	0.0001400
7	hsa-miR-15a	3.58	0.0001929
8	hsa-miR-26b	5.99	0.0003248
9	hsa-miR-455-3p	5.18	0.0003526
10	hsa-let-7f	6.49	0.0003598
11	hsa-miR-27a	2.83	0.0005683
12	hsa-miR-96	5.08	0.0006193
13	hsa-miR-590-5p	3.00	0.0007849
14	hsa-miR-28-5p	4.68	0.0008894
15	hsa-let-7a	4.16	0.0009845

P<0.001.

**Table II:** Target genes of differentially expressed miRNA.

n	hsa-let-7a	hsa-let-7f	hsa-miR-15a	hsa-miR-15b	hsa-miR-21	hsa-miR-26b	hsa-miR-27a	hsa-miR-28-5p	hsa-miR-96	hsa-miR-146b-5p	hsa-miR-424	hsa-miR-590-5p
1	ABCC5	ABCC5	ABCC5	ABCC5	SMAD7	APC	BMI1	HOXA1	CAV1	SMAD4	ABCC5	PLAG1
2	ADRB2	ADRB2	ADRB2	ADRB2	CDC25A	ATM	CD28		HOXA5		ADRB2	SOX2
3	CASP3	CASP3		BCL2	PDCD4	CDK6	CD44		KRAS		BDNF	STAT3
4	CCR7	CCR7	BDNF	BDNF	PDCD4	GSK3B	CYP1B1		MTMR3		CD28	WWP1
5	CDC25A	CDC25A	CCND1	CCND1	PLAG1	HMGA2	EGFR		PAK1		CDC25A	
6	COL1A1	COL1A1	CD28	CD28	SOX2	HOXA5	HOXA10		RAC1		CYP26B1	
7	ERCC6	ERCC6	CDC25A	CDC25A	STAT3	HOXA9	ING5		SMAD7		FGF2	
8	FASLG	HMGA2	CYP26B1	CYP26B1	STAT3	MAP2	KRAS		ZIC2		FGFR1	
9	HMGA2	HOXA1	FGF2	FGF2	STAT3	PIM1	MSI1				HOXA10	
10	HOXA1	HOXA9	FGFR1	FGFR1	WWP1	PTEN	PLAG1				MYB	
11	HOXA9	IGF1R	HMGA2	HOXA10		SENP5	SFRP1				PLAG1	
12	IGF1R	IGF2BP3	HOXA10	MYB		SMAD4					SMAD3	
13	IGF2BP3	IKBKE	IGF2R	PDCD4							SMAD7	
14	IKBKE	IL10	MYB	PIM1							TGFBR3	
15	IL10	SENP5	PDCD4	PLAG1							WWP1	
16	KRAS		PIM1	SMAD3								
17	PAK1		PLAG1	SMAD7								
18	SENP5		SMAD3	TGFBR3								
19			SMAD7	WWP1								
20			TGFBR3									
21			WWP1									

ABCC5, ATP-binding cassette, sub-family C; SMAD, SMAD family member 4; APC, adenomatosis polyposis coli; BMI1, BMI1 polycomb ring finger oncogene; HOXA, homeobox A cluster; CAV1, caveolin 1; PLAG1, pleiomorphic adenoma gene 1; ADRB2, adrenoceptor β2; CDC25A, cell division cycle 25A; ATM, ataxia telangiectasia mutated; SOX2, SRY (sex determining region Y)-box 2; CASP3, caspase 3, apoptosis-related cysteine peptidase; BCL2, B-cell lymphoma 2; PDCD4, programmed cell death 4; CDK6, cyclin-dependent kinase 6; KRAS, Kirsten rat sarcoma viral oncogene homolog; BDNF, brain-derived neurotrophic factor; STAT3, signal transducer and activator of transcription 3; CCR7, chemokine receptor 7; GSK3B, glycogen synthase kinase 3β; CYP1B1, cytochrome P450, family 1, subfamily B, polypeptide 1; MTMR3, myotubularin related protein 3; WWP1, WW domain containing E3 ubiquitin protein ligase 1; CDC25A, cell division cycle 25A; CCND1, cyclin D1; PLAG1, pleiomorphic adenoma gene 1; HMGA2, high mobility group AT-hook 2; EGFR, epidermal growth factor receptor; PAK1, p21 protein (Cdc42/Rac)-activated kinase 1; COL1A1, collagen, type I, α1; RAC1, ras-related C3 botulinum toxin substrate 1; CYP26B1, cytochrome P450, family 26, subfamily B, polypeptide 1; ERCC6, excision repair cross-complementation group 6; ING5, inhibitor of growth family, member 5; FGF2, fibroblast growth factor 2; FASLG, Fas ligand (TNF superfamily, member 6); MAP2, microtubule associated protein 2; ZIC2, Zic family member 2; FGFR1, fibroblast growth factor receptor 1; PIM1, pim-1 oncogene; MSI1, musashi RNA-binding protein 1; PTEN, phosphatase and tensin homolog; MYB, v-myb avian myeloblastosis viral oncogene homolog; IGF1R, insulin-like growth factor 1 receptor; SENP5, sentrin specific peptidase 5; SFRP1, secreted frizzled-related protein 1; IGF2BP3, insulin-like growth factor 2 mRNA binding protein 3; IKBKE, inhibitor of κ light polypeptide gene enhancer in B-cells, kinase epsilon; IGF2R, insulin-like growth factor 2 receptor; IL10, interleukin 10; TGFBR3, transforming growth factor, β receptor III.

**Table III:** Gene Ontology analysis of the 10 most significant target genes (False discovery rate, P<0.05).

n	Description	P-value	Genes in test set
1	Fibroblast growth factor receptor signaling pathway	0.000004	CEP5, FGF2, FGFR3 and FGFR4
2	Transmembrane receptor protein tyrosine kinase signaling pathway	0.000087	CEP57, FGF2, FGFR3, FGFR4 and IGF1R
3	Enzyme-linked receptor protein signaling pathway	0.000440	CEP57, FGF2, FGFR3, FGFR4 and IGF1R
4	Phosphate metabolic process	0.003100	FGFR2, FGF2, FGFR2, FGF4, IGF1R, SDC3
5	Phosphorus metabolic process	0.003100	FGFR2, FGF2, FGFR2, FGF4, IGF1R, SDC3
6	Phosphorylation	0.009800	FGF2, FGFR3, FGFR4, IGF1R
7	Positive regulation of cell proliferation	0.009800	FGF2, FGFR3, FGFR4, IGF1R
8	Cell surface receptor-linked signal transduction	0.011000	CEP57, IGF2BP3, FGF2, FGFR3, FGFR4, IGF1R and PLAUR
9	Wound healing	0.018000	CD44, FGF2 and FN1
10	Response to wounding	0.019000	CD44, IGF2BP3, FGF2 and FN1

CEP, caenorhabditis elegans; FGF, fibroblast growth factor; FGFR, fibroblast growth factor receptor; IGF1R, insulin-like growth factor 1 receptor; SDC3, syndecan-3; IGF2BP3, insulin-like growth factor 2 mRNA binding protein 3; PLAUR, plasminogen activator urokinase receptor; FN1, fibronectin 1.
